# The Combination of Resveratrol and Quercetin Attenuates Metabolic Syndrome in Rats by Modifying the Serum Fatty Acid Composition and by Upregulating SIRT 1 and SIRT 2 Expression in White Adipose Tissue

**DOI:** 10.1155/2015/474032

**Published:** 2015-11-01

**Authors:** Ana Elena Peredo-Escárcega, Verónica Guarner-Lans, Israel Pérez-Torres, Sergio Ortega-Ocampo, Elizabeth Carreón-Torres, Vicente Castrejón-Tellez, Eulises Díaz-Díaz, María Esther Rubio-Ruiz

**Affiliations:** ^1^Department of Physiology, Instituto Nacional de Cardiología “Ignacio Chávez”, 14080 Mexico City, Mexico; ^2^Department of Pathology, Instituto Nacional de Cardiología “Ignacio Chávez”, 14080 Mexico City, Mexico; ^3^Department of Molecular Biology, Instituto Nacional de Cardiología “Ignacio Chávez”, 14080 Mexico City, Mexico; ^4^Department of Reproductive Biology, Instituto Nacional de Ciencias Médicas y de la Nutrición “Salvador Zubirán”, 14080 Mexico City, Mexico

## Abstract

Resveratrol (RSV) and quercetin (QRC) modify energy metabolism and reduce cardiovascular risk factors included in the metabolic syndrome (MetS). These natural compounds upregulate and activate sirtuins (SIRTs), a family of NAD-dependent histone deacetylases. We analyzed the effect of two doses of a commercial combination of RSV and QRC on serum fatty acid composition and their regulation of SIRTs 1–3 and PPAR-*γ* expression in white adipose tissue. MetS was induced in Wistar rats by adding 30% sucrose to drinking water for five months. Rats were divided into control and two groups receiving the two different doses of RSV and QRC in drinking water daily for 4 weeks following the 5 months of sucrose treatment. Commercial kits were used to determine serum parameters and the expressions of SIRTs in WAT were analysed by western blot. In MetS rats body mass, central adiposity, insulin, triglycerides, non-HDL-C, leptin, adiponectin, monounsaturated fatty acids (MUFAs), and nonesterified fatty acids (NEFAs) were increased, while polyunsaturated fatty acids (PUFAs) and HDL-C were decreased. SIRT 1 and SIRT 2 were downregulated, while PPAR-*γ* was increased. RSV + QRC administration improved the serum health parameters modified by MetS and upregulate SIRT 1 and SIRT 2 expression in white abdominal tissue in MetS animals.

## 1. Introduction

Metabolic Syndrome (MetS) is a complex and heterogeneous disease which is actually considered as an epidemic. MetS groups several cardiometabolic risk factors including abdominal obesity, hyperglycemia, dyslipidemia, insulin resistance, inflammation, and high blood pressure which predispose to the development of type-2 diabetes and cardiovascular diseases [1].

Several factors are involved in the development of MetS which are linked to adipose tissue dysfunction, one of them being the circulating free fatty acids (FFA). In a MetS model developed by our group, we have found alterations in serum lipid composition, that is, high levels of nonesterified fatty acids (NEFAs) and of monounsaturated fatty acids (MUFAs) which have been proposed as contributors of the acquisition of insulin resistance and hypertension [2–4]. Moreover, FFA and their derivatives trigger physiological responses such as adipogenesis and adipokine secretion [5].

Flavonoid intake is positively associated with a decrease in the incidence of metabolic and obesity-related disorders. Resveratrol (RSV) (3,4′,5-trihydroxystilbene) is a phytoalexin found in the skin and seeds of grapes and red wine. RSV may protect against diet-induced obesity and metabolic diseases such as hepatic steatosis and insulin resistance [6]. Quercetin (QRC) (3,5,7,3′,4′-pentahydroxyflavone) is a polyphenolic flavonoid compound present in onions, broccoli, tomatoes, apples, and berries and it possesses antioxidant, anti-inflammatory, and antiatherogenic properties including hepatoprotection [7]. RSV and QRC could be promising therapeutic agents acting as sirtuin activators. They have shown benefic effects for the treatment of metabolic diseases such as obesity and MetS.

The sirtuin (SIRT) family of NAD^+^-dependent protein deacetylases and ADP-ribosyltransferases has emerged as an exciting target for cardiovascular disease management since they can impact the cardiovascular system both directly and indirectly by modulating whole body metabolism [8]. Mammals contain seven sirtuins (SIRTs 1–7) that are localized in distinct subcellular compartments. SIRT 1, SIRT 6, and SIRT 7 are found in the nucleus; SIRT 2 is primarily cytosolic; and SIRTs 3–5 are found in mitochondria [9]. In addition to the differences in subcellular localization, the sirtuins are also expressed in varying amounts in different tissues.

QRC is more effective in reducing adipogenesis in preadipocytes, whereas RSV is more effective in inhibiting lipid metabolism in mature adipocytes. Other studies suggest the synergistic effect of both natural compounds to treat metabolic disorders [10, 11]. Moreover, these compounds are now available in tablets on the market.

Thus, the goal of this study was to examine the effect of the commercial mixture of RSV and QRC on the serum FA profile and on the SIRTs 1–3 expression in white adipose tissue (WAT).

## 2. Materials and Methods

### 2.1. Animals

All experiments were conducted in accordance with the Institutional Ethical Guidelines.

Weanling male Wistar rats aged 25 days and weighing 50 ± 4 g, *n* = 12 per group were separated into two groups: group 1, control rats (C), given tap water for drinking, and group 2, MetS rats, receiving 30% sugar in their drinking water during 5 months.

One-third of each group of rats (control or MetS) received orally in drinking water or sucrose solution a mixture of RSV and QRC daily for 4 weeks (provided by ResVitalé which contains 20 mg of QRC per 1,050 mg of RSV) in one of the following doses: (1) RSV + QRC 10 mg/kg/day–0.19 mg/Kg/day (RSV 10 + QRC 0.19); and (2) RSV + QRC 50 mg/kg/day–0.95 mg/Kg/day (RSV 50 + QRC 0.95). Groups without RSV + QRC treatment only received the vehicle in which the natural compounds were dissolved. The mixture of RSV and QRC was previously dissolved in 1 mL ethanolic solution (20%).

All animals were fed Purina 5001 rat chow (Richmond, IN)* ad libitum*, which provides 14.63 KJ/g with 23% protein, 12% fat, and 65% carbohydrate, and were kept under controlled temperature and a 12 : 12-hour light-dark cycle.

Systolic arterial blood pressure was measured in conscious animals using the tail cuff method as described previously [12].

### 2.2. Blood Samples

At the end of experimental period and after an overnight fasting (12 h), the animals were killed by decapitation and blood was collected. Serum was isolated by centrifugation and stored at –70°C until needed. Serum insulin, adiponectin, and leptin were determined using commercial radioimmunoassay (RIA) kits specific for rat (Linco Research Inc., Missouri, USA); the sensitivity was of 0.1 ng/mL; and intra- and interassay coefficients of variation were 5%, 10%, and 10%, respectively. Glucose concentration was assayed using an enzymatic Kit SERA-PAK^R^ Plus (Bayer Corporation, Sées, France).

Total cholesterol (TC) and plasma triglyceride concentrations were measured using commercial enzymatic assays (RANDOX Laboratories, UK). The high-density lipoprotein (HDL) cholesterol content was determined in the bottom fraction obtained after ultracentrifugation of plasma at density of 1.063 g/mL for 2.5 h at 100,000 rpm (Beckman optima TLX) [13, 14]. The non-HDL-C is defined as the difference between the values of TC and HDL-C and includes LDL-C, IDL, and VLDL. Recently non-HDL-C has become a commonly used marker for a blood lipid pattern associated with increased risk of heart disease.

The homeostasis model assessment of insulin resistance (HOMA-IR) was used as the physiological index of insulin resistance. The HOMA-IR was calculated from the fasting glucose and insulin concentrations by the following formula: (insulin (*μ*U/mL) × glucose (in mmol/L)/22.5) [14].

### 2.3. WAT Homogenate

Abdominal WAT was removed and weighed. The samples were immediately frozen in liquid nitrogen and stored at −70°C for later analysis. Frozen WAT samples were homogenized (25% w/v) in a lysis buffer pH = 8 (25 mM HEPES, 100 mM NaCl, 15 mM imidazole, 10% glycerol, and 1% Triton X-100) and protease inhibitor cocktail [14]. The WAT homogenate was centrifuged at 19,954 g for 10 min at 4°C; the supernatant was separated and stored at −70°C. The protein concentration of each sample was measured using the Bradford method [15].

### 2.4. SIRT 1, SIRT 2, SIRT 3, and PPAR-*γ* Expression

Protein expression was examined by Western blot analysis. A total of 50 *μ*g protein was separated by SDS-PAGE (12% polyacrylamide gel) and transferred to a PVDF membrane. The blots were blocked for 3 hours at room temperature with Tris buffer solution (TBS) containing 5% nonfat dry milk and 0.05% Tween 20. The membranes were incubated overnight at 4°C with rabbit primary polyclonal antibodies (SIRT 1, Santa Cruz Biotechnology, Santa Cruz, CA; SIRT 2, SIRT 3, and PPAR-*γ*, Abcam) at a final dilution of 1 : 1000. Then, the membranes were incubated for 2 h at room temperature with a secondary antibody (goat anti-rabbit horseradish peroxidase conjugated, dilution 1 : 10,000, Santa Cruz Biotechnology, Santa Cruz, CA). After incubation, the blots were visualized using a chemiluminescence kit (Immobilon Western, Millipore, MA, USA). Blots were stripped and reincubated with monoclonal *α*-actin antibody as control. Images from films were digitally acquired by GS-800 densitometer with the Quantity One software (Bio-Rad). The values of each band density are expressed as arbitrary units (AU).

### 2.5. Total Fatty Acid and Nonesterified Fatty Acids Lipid Extraction

Fatty acids (FA) and NEFAs were extracted and identified by gas liquid chromatography, from serum (100 *μ*L) and from the administrated RSV + QRC commercial mixture (50 *μ*g), using the method described previously [4].

### 2.6. Statistical Analysis

Results were expressed as mean ± standard error of the mean (SEM). For multiple comparisons, we applied one-way analysis of variances (ANOVA) using the SigmaPlot 11 program. Differences were considered significant when the *P* value was <0.05.

## 3. Results

Table 1 summarizes the characteristics of the groups of rats used. Experimental animals developed MetS characterized by hypertension, central adiposity, hyperinsulinemia, and insulin resistance (HOMA-IR). Leptin and adiponectin concentrations were significantly higher in the MetS than in control rats. In MetS rats, the treatment with RSV + QRC (both doses) prevented the increase in body weight and significantly decreased the central adiposity; however, leptin concentrations remained high when compared to controls. A tendency towards lower values was observed with the high dose in adiponectin concentrations. Systolic arterial pressure diminished in the MetS RSV-QRC-treated group in a dose-dependent manner.

RSV + QRC significantly reduced insulin concentration in MetS rats and restored HOMA-IR. No differences were found between both doses. Fasting serum glucose levels were not significantly different among the groups. RSV + QRC supplementation did not alter significantly any parameters in control group (Table 1).

Table 2 shows the lipidic profile of both MetS and control animals. MetS showed dyslipidemia (high levels of triglycerides and non-HDL-C and low levels of HDL-C). RSV + QRC significantly reduced the concentration of triglycerides. The high dose was able to diminish the amount of non-HDL-C in experimental group. Although a statistical significance was only present with the low dose in HDL-C, a clear tendency towards increased values was found with the high dose in the MetS group.

In the control group, only the non-HDL-C was significantly decreased with RSV + QRC administration (Table 2). No changes were observed in TC content among the groups.

The FA composition (%) of serum from control and MetS rats is shown in Table 3. Seric concentrations of palmitoleic acid, oleic acid, and MUFA were significantly increased, while stearic and PUFA decreased in MetS rats in comparison to control rats. The treatment with RSV + QRC restored the levels of oleic acid (by the highest RSV + QRC dose) and PUFA (with both doses). In the control group, the treatment with RSV + QRC increased the levels of arachidonic and PUFA in a dose-dependent way.

Table 4 shows the results corresponding to the NEFA present in serum from the six groups studied. NEFAs such as MUFA and palmitoleic and oleic acids were significantly increased in MetS when compared to control animals. SFA, PUFA, stearic, linoleic, and arachidonic levels are diminished in MetS in comparison to those in the controls. In MetS rats, both doses of RSV + QRC increased stearic acid and the highest dose of RSV + QRC significantly diminished palmitoleic acid content. In the control group, the treatment with the natural compounds had no effect.

Additionally, we analyzed the FA composition of the RSV + QRC administrated and we found that linoleic, oleic, and palmitic acids were the most abundant FA (37.8 ± 2.1%, 22.1 ± 1.0%, and 21.6 ± 1.1%, resp.). Stearic (11.9 ± 0.9%), palmitoleic (3.8 ± 0.6%) and arachidonic (2.8 ± 0.3%) acids also were present (data not shown).

To address the effect of RSV + QRC administration on the expression of SIRTs in WAT, we performed immunoblotting analyses.

**Figure 1:**
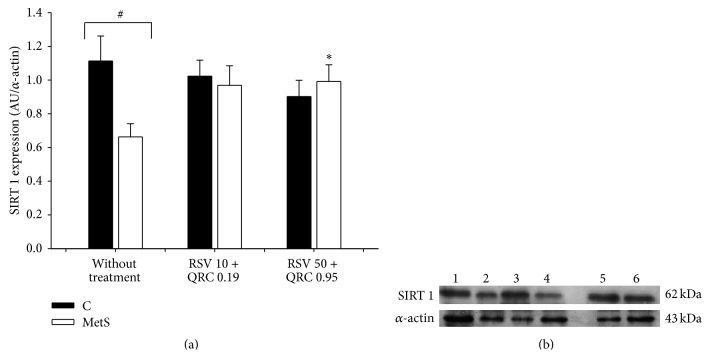
RSV plus QRC leads to SIRT 1 expression in WAT from MetS rats. (a) Protein expression, data represent mean ± SEM (*n* = 6 per group). ^#^ *P* < 0.05; ^*∗*^ *P* < 0.05 against MetS without treatment. (b) Representative Western blot analysis. Line 1: control without treatment; line 2: control treated with RSV 10 + QRC 0.19; line 3: control treated with RSV 50 + QRC 0.95; line 4, MetS without treatment; line 5: MetS treated with RSV 10 + QRC 0.19; line 6: MetS treated with RSV 50 + QRC 0.95.

The data in Figures 1, 3, and 4 show SIRT 1 (62 KDa), SIRT 2 (43 KDa), and SIRT 3 (28 KDa) levels, respectively, of control and MetS rats treated with RSV + QRC. The expression of SIRT 1 and SIRT 2 in MetS rats was reduced when compared to control rats. When assessing the effect of RSV + QRC treatment in MetS group, we observed a clear tendency towards increased values of SIRT 1 expression with the lowest dose and a significant increase with RSV 50 + QRC 0.95 (Figures 1(a) and 1(b)). There was a significant increase in SIRT 2 expression with both doses of RSV + QRC (Figures 3(a) and 3(b)). There was no significant change in SIRT 1 and SIRT 2 expression in control rats treated with RSV + QRC.

SIRT 3 expression in WAT was similar in control and MetS rats (Figures 4(a) and 4(b)). Although SIRT 3 expression levels were not significantly modified, a tendency towards reduced values was observed in MetS animals treated with the highest dose. In control rats, the opposite effect was observed and the level of expression of the protein was significantly higher than in MetS rats.

**Figure 2:**
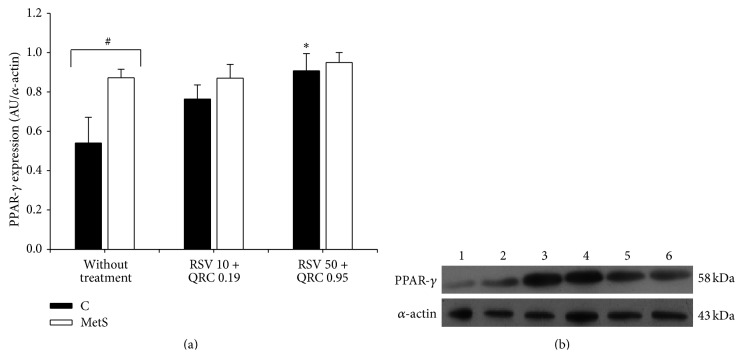
Effect of RSV + QRC administration on PPAR-*γ* expression in WAT from control and MetS rats. (a) Protein expression, data represent mean ± SEM (*n* = 6 per group). ^#^ *P* < 0.05; ^*∗*^ *P* < 0.05 against control without treatment. (b) Representative Western blot analysis. Line 1: control without treatment; line 2: control treated with RSV 10 + QRC 0.19; line 3: control treated with RSV 50 + QRC 0.95; line 4, MetS without treatment; line 5: MetS treated with RSV 10 + QRC 0.19; line 6: MetS treated with RSV 50 + QRC 0.95.

We also evaluated the effect of RSV + QRC on PPAR-*γ* expression, a target of SIRT 1 (Figures 2(a) and 2(b)). As expected, the levels of PPAR-*γ* were significantly increased in MetS rats in comparison to control animals. The administration of both of the doses of RSV + QRC did not significantly modify PPAR-*γ* expression. In contrast, the highest dose of RSV + QRC significantly increased PPAR-*γ* expression in the control group.

## 4. Discussion

MetS is actually considered as an epidemic and is a complex and heterogeneous disease. Therapeutic tools used to control MetS include lifestyle changes (increases in physical activity and caloric restriction), pharmacological agents, and natural compounds. Although several studies have shown that the RSV and/or QRC that are present in plants and fruits have beneficial effects on metabolic disorders by regulating sirtuin expression and activity, the effect of these compounds on changes in serum FFA still remain unclear. In the present work, we analyzed the effects of two doses of a combination of RSV and QRC on body fat, serum parameters, SIRTs 1–3, and PPAR-*γ* expression in a rat model of MetS.

MetS rats exhibited increased body weight, central adiposity, hypertension, insulin resistance, and elevated circulating levels of adiponectin and leptin (Table 1). These results are in accordance with our previous report [14]. Several studies have demonstrated beneficial effects of RSV and QRC reducing body fat and improving insulin sensitivity [16]. In MetS rats, both of the doses of RSV + QRC treatment tested in this paper were equally efficient in reducing body weight, blood pressure, and insulin levels without having an effect on the concentration of adiponectin and leptin.

In the MetS group, RSV + QRC treatment attenuated the increase in blood pressure in a dose-dependent manner (14% and 22% by RSV 10 + QRC 0.19 and RSV 50 + QRC 0.95, resp.) (Table 1). The antihypertensive effect of RSV and QRC may be due to activation of several mechanisms which have already been described and that include increased NO availability caused by the elevation of NOS activity and by a decrease in oxidative stress and inflammation [17–19].

Our MetS model had high circulating levels of leptin and adiponectin compared to control animals, suggesting the presence of resistance to these adipokines as previously reported (Table 1) [14]. A possible mechanism by which polyphenols might act is by regulating adipokine levels and their intracellular signaling mechanisms [10, 18, 20]. In the present study, the supplementation with RSV + QRC had no effect upon the leptin concentration and only caused a slight decrease in adiponectin levels (13% by RSV 50 + QRC 0.95). This discrepancy with other reports might be due to the different administration periods used. Possibly, if we increased the duration of the polyphenol administration period, a difference in adipokine levels might become evident.

In the control rats, none of the parameters studied was affected by either dose of RSV + QRC (Table 1). These results are consistent with data from RSV studies conducted in lean metabolically normal rodents and in human subjects [21].

MetS rats developed dyslipidemia with decreased levels of HDL-C and increased levels of non-HDL-C and triglycerides when compared to control animals (Table 2). In the MetS group, the highest dose of RSV + QRC was effective in reducing triglycerides and non-HDL-C, while, in control animals, only the highest dose was able to reduce non-HDL-C concentration. Our results on the improvement of dyslipidemia with the RSV + QRC treatment are in accordance with those published by other authors who used natural compounds separately in other models of obesity or in isolated adipocytes [10, 18, 22]. The importance of testing these compounds in our model resides in the fact that our model resembles the appearance of MetS by the ingestion of high sucrose levels in a similar way as it happens in humans who consistently ingest sucrose in sweetened beverages.

Table 3 shows seric FA composition in both control and MetS rats. Circulating PUFA levels were decreased in MetS rats when compared to controls and these data positively correlate with the increase in central adiposity present in this group. Both doses of RSV + QRC increased PUFA concentrations in MetS rats in the same proportion (33%), while in control rats the increase in PUFA concentration seemed to be dose-dependent (15% and 35% by RSV 10 + QRC 0.19 and RSV 50 + QRC 0.95, resp.). Regarding this aspect, Rodriguez-Cruz et al. [23] reported that high levels of PUFAs are negative regulators of lipogenesis. PUFAs may function as activators/ligands of PPAR-*γ* limiting hyperplasia and hypertrophy of adipose tissue [24].

There are many evidences indicating that dietary MUFAs reduce key risk factors for MetS. However, as far as we know, there are very few studies that show the benefic effects of RSV + QRC on the seric profile of MetS rat models. In serum from MetS rats, MUFAs such as palmitoleic and oleic acids were increased when compared to those of control animals (55% and 50%, resp.) (Table 3). RSV 50 + QRC 0.95 significantly diminished oleic acid concentration without having an effect on palmitoleic levels. The decrease on circulating oleic acid by RSV + QRC may be related to the attenuation of high blood pressure [4].

There is a tendency to increase seric arachidonic acid concentration with the RSV + QRC treatment (with and without significant difference, resp.) in the control and MetS groups (Table 3). Some authors have reported that RSV and QRC may modulate arachidonic acid release and metabolism due to their anti-inflammatory activity [25–27]; however, future studies need to be undertaken to examine the effect of RSV + QRC on arachidonic acid metabolism and its contribution to diminished blood pressure and other parameters in our rat MetS model.

Circulating levels of NEFAs such as oleic and palmitoleic acid were higher in MetS when compared to their levels in control rats (Table 4). These fatty acids were more abundant than triglycerides, cholesterol esters, and phospholipids [28]. Thus, our results show that central adiposity leads to an important increase in NEFA and triglyceride production in MetS animals. High concentrations of NEFA are related to oxidative stress, hypertension, dyslipidemia, and insulin resistance [3, 4, 29]. The RSV + QRC treatment produced a slight decrease in oleic and palmitoleic levels (3% and 16%, resp.). Moreover, the decrease in fat accumulation was accompanied by a decrease in the concentration of free fatty acids with a concomitant decrease in triglyceride concentrations. Our results were consistent with other studies which indicated that RSV and QRC significantly suppressed the serum NEFA levels; however, in the present study we administer the combination of flavonoids and identified each NEFA, which had not been previously reported [19, 30, 31].

Several reports have shown that RSV and QRC are sirtuin activators; therefore, we investigated the effect of RSV + QRC on SIRTs 1–3 expression in WAT from control and MetS rats.  Figure 1 shows that SIRT 1 was underexpressed in MetS rats and that the treatment with both doses of RSV + QRC restored SIRT 1 expression. Our data are in accordance with several animal studies that have provided strong evidence on the positive effect of RSV and QRC upregulating SIRT 1 in different models. SIRT 1 is an important regulator of hepatic glucose metabolism; it improves insulin signaling and promotes fatty acid metabolism [32, 33]. However, in the present work, SIRT 1 overexpression did not have a significant effect on adipokine secretion.

SIRT 1 regulates the pathway of cellular energy metabolism, controlling PGC1-*α*, p53, forkhead transcription factors (FOXO), p300, and PPAR-*γ* which plays an important role in the induction of cellular differentiation of adipocytes and in the regulation of lipid metabolism. Floyd et al. [34] reported that resveratrol modulates PPAR-*γ* protein levels and transcriptional activity in 3T3-L1 adipocytes. Moreover, SIRT 1 represses PPAR-*γ* in WAT by attaching to its cofactor's nuclear receptor corepressor (NCoR) [35]. In this study, when the expression of PPAR-*γ* was measured, its levels were higher in WAT from MetS rats than in control subjects (Figure 2). This result had been previously reported by our group [14]. The treatment with RSV + QRC had no effect on PPAR-*γ* expression on MetS group, while in the control group RSV 50 + QRC 0.95 significantly increased PPAR-*γ* levels. Although the treatment with these concentrations of polyphenols had no effect on the PPAR-*γ* expression in MetS animals, RSV + QRC could be regulating PPAR-*γ* activity. To further clarify this point, it would be important to evaluate the effect of RSV + QRC administration on gene expression of transcriptional targets of PPAR-*γ* and on other adipogenic markers such as C/EBP*β* and SREBP1c. Our results agree with those of a previous study that reported that QRC did not modify PPAR-*γ* expression in 3T3-L1 preadipocytes [10].

**Figure 3:**
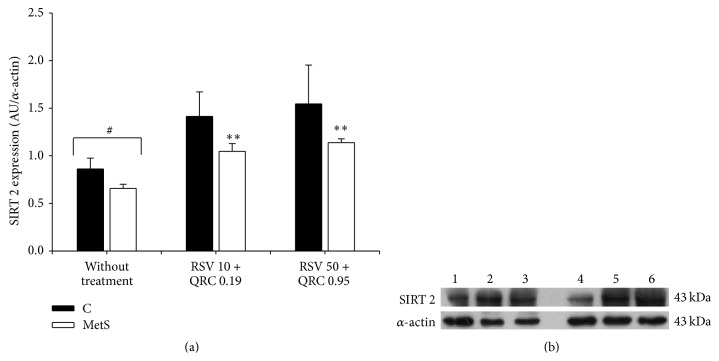
RSV plus QRC leads to SIRT 2 expression in WAT from MetS rats. (a) Protein expression, data represent mean ± SEM (*n* = 6 per group). ^#^ *P* < 0.05; ^*∗∗*^ *P* < 0.01 against MetS without treatment. (b) Representative Western blot analysis. Line 1: control without treatment; line 2: control treated with RSV 10 + QRC 0.19; line 3: control treated with RSV 50 + QRC 0.95; line 4, MetS without treatment; line 5: MetS treated with RSV 10 + QRC 0.19; line 6: MetS treated with RSV 50 + QRC 0.95.

SIRT 2 is reported to be the most abundant sirtuin in adipocytes, in white and brown adipose tissue [36]. As expected, SIRT 2 expression was decreased in WAT from MetS rats in comparison to control rats, which might promote fat accumulation (Figure 3). RSV + QRC induced SIRT 2 expression in a dose-dependent manner (59% and 73% by RSV10 + QRC 0.19 and RSV50 + QRC 0.95, resp.), while in the control group it had no effect. The effect of RSV + QRC on SIRT 2 expression agrees with the results previously reported by Gregory [37].

**Figure 4:**
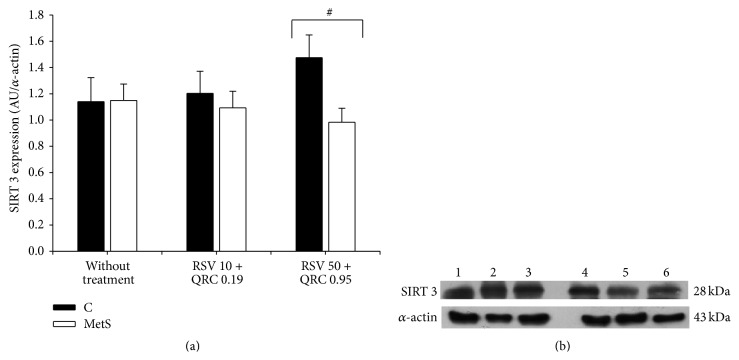
Effect of RSV + QRC administration on SIRT 3 expression in WAT from control and MetS rats. (a) Protein expression, data represent mean ± SEM (*n* = 6 per group). ^#^ *P* < 0.05. (b) Representative Western blot analysis. Line 1: control without treatment; line 2: control treated with RSV 10 + QRC 0.19; line 3: control treated with RSV 50 + QRC 0.95; line 4, MetS without treatment; line 5: MetS treated with RSV 10 + QRC 0.19; line 6: MetS treated with RSV 50 + QRC 0.95.

SIRT 3 is the major mitochondrial deacetylase regulating mitochondrial metabolism, adaptive thermogenesis, energy homeostasis, and apoptosis and it is decreased in obese mice [38, 39]. Moreover, SIRT 3 plays an important role in adaptive thermogenesis of brown adipose tissue regulating UCP-1, PGC-1*α*, cytochrome c oxidase, and ATP synthase expression. When we analyzed the expression of SIRT 3, we found that WAT from control and MetS rats expressed SIRT 3 in the same proportion and its expression was not significantly modified by the RSV + QRC treatment (Figure 4). However, there was a tendency to an increase in the SIRT 3 levels in control rats when compared to MetS animals treated with the highest dose of RSV + QRC (Figure 4). The precise reason for this discrepancy is unclear but it would be interesting to evaluate the effect of RSV + QRC on SIRT 3 activity.

In conclusion, our data suggest that RSV + QRC (particularly RSV50 + QRC 0.95) influences adipose tissue mass and function in a way that may positively interfere with the development of MetS. This effect may be associated with an increase in PUFA and with a decrease in circulating levels of MUFA and NEFA. Moreover, RSV + QRC upregulates SIRT 1 and SIRT 2 expression in WAT.

## Figures and Tables

**Table 1:** The effects of RSV + QRC administration on body characteristics and biochemical parameters from control and MetS rats.

	Control			MetS		
	Without treatment	RSV 10 + QRC 0.19 mg/kg/day	RSV 50 + QRC 0.95 mg/kg/day	Without treatment	RSV 10 + QRC 0.19 mg/kg/day	RSV 50 + QRC 0.95 mg/kg/day
Body weight (g)	502.0 ± 19.5	463.8 ± 20.8	514.2 ± 20.9	570.4 ± 13.4^a^	490.8 ± 11.6^c^	489.8 ± 10.5^c^
Central adiposity (g)	6.0 ± 0.7	4.5 ± 0.6	6.3 ± 0.9	12 ± 0.6^a^	11.5 ± 0.9	8.9 ± 1.1^c^
Blood pressure (mm Hg)	103.3 ± 1.0	101.2 ± 2.7	108.4 ± 3.5	140.5 ± 1.0^a^	122.7 ± 3.8^c^	115.5 ± 2.9^c^
Glucose (mg/dL)	119.9 ± 12.2	118.8 ± 13.7	87.9 ± 5.9	121.8 ± 20.3	85.3 ± 9.5	90.7 ± 7.7
Insulin (ng/mL)	0.26 ± 0.02	0.25 ± 0.05	0.18 ± 0.04	0.47 ± 0.04^a^	0.29 ± 0.05^c^	0.23 ± 0.02^c^
HOMA-IR	1.3 ± 0.2	0.61 ± 0.03	0.9 ± 0.12	2.1 ± 0.3^b^	0.9 ± 0.1^c^	0.81 ± 0.1^c^
Leptin (ng/mL)	2.3 ± 0.3	3.3 ± 0.3	2.6 ± 0.1	4.2 ± 0.3^a^	5.2 ± 0.3^d,e^	3.8 ± 0.3^e^
Adiponectin (*μ*g/mL)	3.8 ± 0.2	4.2 ± 0.3	3.7 ± 0.3	6.7 ± 0.3^a^	6.1 ± 0.5^e^	5.8 ± 0.2^e^

Values are mean ± SEM. HOMA-IR: homeostatic model assessment of insulin resistance; *n* = 12; ^a^ *P* < 0.01 MetS without treatment versus control without treatment; ^b^ *P* < 0.05 MetS without treatment versus control without treatment; ^c^ *P* < 0.01 against same group without treatment; ^d^ *P* < 0.01 versus same group with different doses; ^e^ *P* < 0.01 against control with same dose.

**Table 2:** The effects of RSV + QRC administration on serum triglycerides, total cholesterol (TC), HDL-C, and non-HDL-C levels from control and MetS rats.

	Control			MetS		
	Without treatment	RSV 10 + QRC 0.19 mg/kg/day	RSV 50 + QRC 0.95 mg/kg/day	Without treatment	RSV 10 + QRC 0.19 mg/kg/day	RSV 50 + QRC 0.95 mg/kg/day
Triglycerides (mg/dL)	77.8 ± 7.9	71.4 ± 7.4	57.8 ± 9.2	133.7 ± 6.3^a^	103.2 ± 9.7^e^	90.5 ± 5.4^c,e^
TC (mg/dL)	57.6 ± 5.6	55.5 ± 3.4	45.7 ± 1.7	52.3 ± 3.5	56.6 ± 5.7	38.2 ± 4.7
HDL-C (mg/dL)	28.2 ± 2.5	27.1 ± 1.8	28.6 ± 1.6	17.6 ± 1.8^a^	29.1 ± 4.2^c,d^	20.3 ± 2.4
non-HDL-C (mg/dL)	22.8 ± 2.1	29.4 ± 2.7	17.1 ± 0.4^d^	35.2 ± 3.02^a^	27.5 ± 2.2	17.9 ± 2.8^c,d^

Values are mean ± SEM. *n* = 12; ^a^ *P* < 0.01 MetS without treatment versus control without treatment; ^c^ *P* < 0.01 against same group without treatment; ^d^ *P* < 0.01 versus same group with different doses; ^e^ *P* < 0.01 against control with same dose.

**Table 3:** Effect of RSV + QRC administration on seric fatty acid (FA) composition from control and MetS rats.

FA %	Control			MetS		
	Without treatment	RSV 10 + QRC 0.19 mg/kg/day	RSV 50 + QRC 0.95 mg/kg/day	Without treatment	RSV 10 + QRC 0.19 mg/kg/day	RSV 50 + QRC 0.95 mg/kg/day
Palmitic acid	32.7 ± 0.9	31.7 ± 0.6	32.1 ± 1.2	33.5 ± 0.6	32.7 ± 1.1	32.2 ± 1.2
Palmitoleic acid	4.3 ± 0.7	5.6 ± 1.1	4.5 ± 0.8	6.7 ± 0.3^a^	6.2 ± 0.5	7.3 ± 0.6
Stearic acid	22.2 ± 0.9	22.8 ± 0.9	22.6 ± 1.1	19.4 ± 1.1	19.5 ± 1.0	19.8 ± 0.5
Oleic acid	14.3 ± 1.1	13.5 ± 0.6	13.3 ± 0.7	21.9 ± 1.3^a^	22.1 ± 0.4	17.7 ± 1.1^c^
Linoleic acid	13.6 ± 0.8	12.6 ± 0.8	14.1 ± 0.9	10.5 ± 1.2	11.3 ± 0.7	10.3 ± 0.5
*γ*-linoleic acid	0.3 ± 0.03	0.9 ± 0.4	0.4 ± 0.1	0.8 ± 0.3	0.5 ± 0.1	0.3 ± 0.1
Dihomo-*γ*-linoleic acid	1.2 ± 0.5	0.6 ± 0.1	0.6 ± 0.2	0.7 ± 0.4	0.4 ± 0.05	0.8 ± 0.4
Arachidonic acid	5.5 ± 1.1	10.7 ± 1.1^c^	12.1 ± 0.9^c^	6.7 ± 0.9	7.9 ± 0.6	7.3 ± 0.4
SFA	59.9 ± 1.4	55.9 ± 2.0	54.7 ± 1.7	54.0 ± 1.5	52.3 ± 1.5	52.7 ± 1.7
MUFA	17.8 ± 1.0	19.0 ± 1.4	17.8 ± 0.8	28.6 ± 1.6^a^	27.6 ± 0.5	25.6 ± 1.4
PUFA	20.3 ± 1.6	23.5 ± 1.2^c^	27.2 ± 1.6^c,d^	15.3 ± 0.8^a^	20.1 ± 1.2^c^	19.2 ± 0.5^c^

SFA: saturated fatty acid, MUFA: monounsaturated fatty acid, and PUFA: polyunsaturated fatty acid. Data are mean ± SEM. *n* = 12; ^a^ *P* < 0.01 MetS without treatment versus control without treatment; ^c^ *P* < 0.01 against same group without treatment; ^d^ *P* < 0.05 versus same group with different dose.

**Table 4:** Effect of RSV + QRC administration on seric nonesterified fatty acids (NEFAs) composition from control and MetS rats.

NEFAs %	Control			MetS		
	Without treatment	RSV 10 + QRC 0.19 mg/kg/day	RSV 50 + QRC 0.95 mg/kg/day	Without treatment	RSV 10 + QRC 0.19 mg/kg/day	RSV 50 + QRC 0.95 mg/kg/day
Palmitic acid	34.9 ± 1.3	32.5 ± 1.4	33.6 ± 0.9	33.6 ± 0.8	32.8 ± 0.9	33.6 ± 0.7
Palmitoleic acid	6.2 ± 0.9	5.3 ± 0.8	5.1 ± 0.9	13.1 ± 0.8^a^	11.6 ± 0.5	10.9 ± 0.5^c^
Stearic acid	22.2 ± 0.5	24.1 ± 0.7	23.1 ± 0.8	16.2 ± 0.5^a^	19.2 ± 1.1^c^	19.8 ± 0.5^c^
Oleic acid	17.8 ± 1.0	19.4 ± 1.0	18.6 ± 0.9	22.5 ± 0.9^a^	23.0 ± 0.5	21.9 ± 0.5
Linoleic acid	13.7 ± 1.4	14.3 ± 0.6	13.9 ± 0.9	8.8 ± 0.3^b^	8.1 ± 0.4	9.0 ± 0.4
*γ*-linoleic acid	1.3 ± 0.2	1.4 ± 0.3	1.4 ± 0.3	1.6 ± 0.3	1.1 ± 0.2	1.2 ± 0.3
Dihomo-*γ*-linoleic acid	0.7 ± 0.08	0.8 ± 0.1	0.6 ± 0.07	0.5 ± 0.1	0.4 ± 0.05	0.6 ± 0.07
Arachidonic acid	3.1 ± 0.5	2.4 ± 0.2	3.9 ± 0.4	2.1 ± 0.3^b^	3.1 ± 0.7	2.5 ± 0.3
SFA	57.2 ± 1.6	56.7 ± 1.6	56.7 ± 0.9	49.8 ± 0.9^a^	52.0 ± 1.05	53.4 ± 1.1
MUFA	22.6 ± 1.4	24.6 ± 1.1	23.7 ± 0.6	36.2 ± 0.9^a^	34.3 ± 0.6	32.4 ± 0.8
PUFA	17.6 ± 0.9	18.7 ± 0.6	19.8 ± 1.3	13.5 ± 0.7^b^	13.7 ± 1.1	14.1 ± 1.1

SFA: saturated fatty acid, MUFA: monounsaturated fatty acid, and PUFA: Polyunsaturated fatty acid. Data are mean ± SEM. *n* = 12; ^a^ *P* < 0.01 MetS without treatment versus control without treatment; ^b^ *P* < 0.05 MetS without treatment versus control without treatment; ^c^ *P* < 0.01 against same group without treatment.
